# CD44v6-O-MWNTS-Loaded Gemcitabine and CXCR4 siRNA Improves the Anti-tumor Effectiveness of Ovarian Cancer

**DOI:** 10.3389/fcell.2021.687322

**Published:** 2021-07-07

**Authors:** Wen Yin, Su-Min Qian

**Affiliations:** Department of Gynecology II, Cangzhou Central Hospital, Cangzhou, China

**Keywords:** ovarian cancer, MWNTs, CD44v6, CXCR4, gemcitabine

## Abstract

Ovarian cancer is one of the most common malignancies of the female reproductive system and the deadliest gynecologic cancer. CXCR4 is expressed in a variety of malignant tumors such as breast, prostate, and ovarian cancers. It is also closely related to the migration, invasion, and metastasis of tumor cells. Carbon nanotubes have great potential for targeted therapy of tumors. CD44v6 is not expressed in normal ovarian tissues but is highly expressed in ovarian epithelial carcinoma. In the present study, we applied small interfering RNA targeting the CXCR4 gene and the clinical treatment gemcitabine and oxaliplatin of ovarian cancer as the therapeutic drug, and organically integrated chemotherapy and gene therapy through carbon nanotubes, and used CD44v6 single chain antibody as the targeting moiety to explore its application in ovarian cancer treatment. Significantly, we successfully synthesized CD44v6-O-MWNTS/Gemcitabine/1,2-dioleoyl-3-trimethylammonium-propane (DOTAP)/siRNA system and the results were observed by transmission electron microscope (TEM) and scanning electron microscope (SEM). CD44v6-O-MWNTS/Gemcitabine/DOTAP was able to fully load siRNA at the ratio of 1:2.5. The carbon nanotubes could protect the siRNA. The drug release analysis showed that O-MWNTS/drug/DOTAP/siRNA was able to effectively release the siRNA, and gemcitabine or oxaliplatin in a time-dependent manner. O-MWNTS/drug/DOTAP/siRNA was able to be effectively uptake by ovarian cancer cells. The cellular uptake of CD44v6-O-MWNTS/drug/DOTAP/siRNA mainly depends on lipid raft-mediated endocytosis. CD44v6-O-MWNTS/drug/DOTAP/siRNA improved the effect of siRNA on the inhibition of ovarian cancer cell viability and the induction of cell apoptosis. The expression of CXCR4 was decreased by CD44v6-O-MWNTS/drug/DOTAP/siRNA in ovarian cancer cells. Tumorigenicity analysis in nude mice showed that CD44v6-O-MWNTS/drug/DOTAP/siRNA significantly repressed the tumor growth of ovarian cancer cells *in vivo*. The levels of Ki-67 and CXCR4 were repressed by CD44v6-O-MWNTS/drug/DOTAP/siRNA in the system. Thus, we concluded that the obtained CD44v6-O-MWNTS could effectively load gemcitabine or oxaliplatin, and CXCR4 siRNA, internalized by cancer cells and realized notable *in vitro* and *in vivo* inhibitory function against ovarian cancer growth. Our study provides a promising nanomaterial for the co-delivery of siRNA and anti-tumor drugs for the therapy of ovarian cancer.

## Introduction

Ovarian cancer is one of the most frequently occurring malignant cancer types among women worldwide (Siegel et al., 2020). Despite an increased survival rate and decreased incidence in recent decades, thanks to the developed surgical operation and chemotherapy, most women at an advanced stage of ovarian cancer present recurrence after chemotherapy and radiotherapy. Moreover, non-selective targeting chemotherapy would lead to severe toxicity in normal organs and cause side effects, as well as a short-term efficacy, which limits its application (Narod, 2016). There is an urgent need for novel approaches to systemic and precise treatment. Knowledge on the genetic features of carcinogenesis has greatly accumulated and leads to the fast development of targeted therapy (Eisenhauer, 2017). Among targeted therapies, small interfering RNAs (siRNAs) have recently gained great attention.

Small interfering RNA is a form of double stranded RNA with 20–25 base pairs that could interact with specific mRNAs and cause their degradation, thus achieving efficient gene targeting (Saw and Song, 2020). The high efficacy and specific targeting features of siRNAs show better advantages over DNA therapy and traditional chemotherapy (van den Brand et al., 2018). The application of siRNAs as a therapeutic agent for cancer treatment faces the major problem of being safely delivered to tumor sites, due to their poor stability in the complicated physical environment such as existing various enzymes, as well as the negative charge which impede the cellular uptake and following trafficking to targets (van den Brand et al., 2018). Hence, the design of proper siRNA delivery systems is a significant and promising research orientation.

In recent years, carbon nanotubes have been widely studied as vectors for drug delivery based on their unique structure and physicochemical property, for flexible the designation of nanomaterials with high biocompatibility (Wong et al., 2013). Moreover, carbon nanotubes-based biomaterials, such as multi-walled carbon nanotubes (MWNTS), show great membrane penetration ability, high loading capacity, low immunogenicity, and could be rapidly excreted, hence they are extensively researched for drug and siRNA delivery in cancer treatment (Wang et al., 2015). For example, Pereira et al. (2015) designed MWNT-liposome hybrids for the delivery of doxorubicin and siRNA targeting Polo-like kinase 1 and realized a reversed chemoresistance in lung cancer cells.

Gemcitabine is a pyrimidine antagonist, that functions through impeding DNA synthesis and the progression from G1 to S phase, and eventually causes cell death (Berg et al., 2019). It is often used for ovarian cancer patients with platinum-resistance, and applied in combination with other target therapies (Berg et al., 2019; Konstantinopoulos et al., 2020). Hence, in this study, we loaded gemcitabine together with siRNA in the novel MWNT delivery system to achieve a better therapeutic effect for ovarian cancer and to improve the targeting ability of these delivery systems, we conjugated the MWNTS to a CD44v6 monoclonal antibody, which could specifically interact with the CD44v6 protein highly expressed on the surface of malignant cancer cells (Todaro et al., 2014). The obtained CD44v6-O-MWNTS could effectively load gemcitabine and siRNA, internalized by cancer cells and realized *in vitro* and *in vivo* inhibitory function against ovarian cancer growth. Our study provides a promising nanomaterial for the co-delivery of siRNA and anti-tumor drugs in the treatment of ovarian cancer.

## Materials and Methods

### Materials and Cell Lines

Pristine MWNTs (purity at 95%, length between 0.5 and 2.0 um, and diameter between 4 and 6 nm) were obtained from XFNANO Materials (Nanjing, China). CD44v6 monoclonal antibody was purchased from Abcam (Cambridge, MA, United States). Gemcitabine (Gem), oxaliplatin (Oxa), 1,2-dioleoyl-3-trimethylammonium-propane (DOTAP), agarose, coumarin C6, and 3-(4,5-dimethyl-2-thiazolyl)-2,5-diphenyl-2H-tetrazolium bromide (MTT) were purchased from Sigma-Aldrich (United States). Lipofectamine 2000 was obtained from Invitrogen (Carlsbad, CA, United States). The inhibitors used in this study (MβCD, dynasore, chlorpromazine) were purchased from MedChemExpress (MCE, South Brunswick Township, NJ, United States). The ovarian cancer cell line SW626 MG and SKOV-3 were purchased from American Type Cell Culture (ATCC, United States). Small interfering RNA (siRNA) targeting CXCR4 (siCXCR4) was designed and synthesized by RiboBio (Guangzhou, China). Cells were cultured in Dulbecco’s modified eagle’s medium (DMEM, Gibco) added with 1% penicillin and streptomycin (MO, Sigma) and fetal bovine serum (FBS, 10%, Gibco), and maintained in 37°C incubator filled with 5% CO_2_.

### Morphology

The structure and morphology of MWNT were assessed by transmission electron microscope (TEM, FEI, United States) and scanning electron microscope (SEM, Hitachi, Japan). For the TEM experiment, the samples were suspended in deionized (DI) water at a density, dropped in copper grid covered, stained with uranyl acetate, and dried.

### Synthesis of CD44v6 Single Chain Antibody Modified O-MWNTS/Gemcitabine/DOTAP and O-MWNTS/Oxaliplatin/DOTAP

#### Synthesis and Characterization of CD44v6 Single Chain Antibody Modified O-MWNTs (CD44v6-O-MWNTS)

The raw MWNTS (150 mg) and nitric acid (1 M, 200 mL) dilute were stirred at room temperature for 24 h, followed by filtration to obtain purified MWNTS. The purified MWNTS (100 mg) was ultrasonicated for 4 h with 150 mL of 4M concentrated nitric acid, filtered, rinsed with MilliQ, and dried in a vacuum chamber to obtain carboxylated MWNTS (O-MWNTS). The morphology and structure of O-MWNT were observed by TEM and Raman spectra. The Raman spectra were evaluated by a Raman spectroscopy (Horiba 800, Piscataway, NJ, United States) at room temperature. The anti-CD44V6 monoclonal antibody (1 g/L, 100 μL) was put in 900 μL PBS buffer containing EDTA (10 mM, pH 7.2) for 12 h, and subjected to ultrafiltration centrifuge (MWCO, 5 kDa, 3,500 r/min) for 20 min. Next, 2-MEA (6 mg) was added to incubate at 37°C for 90 min. The solution was centrifuged as above, washed with PBS, and repeated three times. Meanwhile, the O-MWNTS (0.06 mL) was mixed with EDC/Sulfo NHS solution (30 μL, 2 g/L), and agitated for 10 min at room temperature to activate the carboxyl functional groups on the surface. Subsequently, the anti-CD44V6 monoclonal antibody was added to react for 24 h. The obtained CD44v6-O-MWNTS was stored at 4°C for further study. The structure of CD44v6-O-MWNTS was determined by Fourier Transform Infrared Spectroscopy (FTIR, AIM8000, Shimadzu, Kyoto, Japan), ^1^H NMR (Mercury Plus 400, Varian, United States), and Raman spectroscopy.

### Preparation and Characterization of CD44v6-O-MWNTS/Gemcitabine/DOTAP and O-MWNTS/Oxaliplatin/DOTAP

Gemcitabine or oxaliplatin (5 mg/ml) and CD44v6-O-MWNTS were dispersed in 1 mL anhydrous ethanol for 60 min by ultrasound, during which, 3 mL PBS was slowly dropped in the solutions. After 1 h of centrifugation, 8 mg PVPK30 and 8 mg granulesten were dissolved in 1 mL MilliQ, and added to the sediment, followed by ultrasonication over 10 times (250 W, 6 s). Then the mixture was centrifuged at 300 rpm for 5 min. The supernatant was collected, mixed with 15 mg DOTAP, and ultrasonicated for 2 h to obtain the final CD44v6-O-MWNTS/Gemcitabine/DOTAP. The different particle size, polydispersity index, and zeta potential of O-MWNT and CD44v6-O-MWNTS/Gemcitabine/DOTAP were measured in deionized (DI) water through dynamic light scattering (DLS) in a Malvern Zetasizer (Nano ZS, Malvern Instruments, United Kingdom).

### Characterization of CD44v6-O-MWNTS/Gemcitabine/DOTAP

#### siRNA-Loading Capacity

CD44v6-O-MWNTS/Gemcitabine/DOTAP was mixed with siCXCR4 at different N/P ratios (1.25:1, 2.5:1, 5:1, 7.5:1, and 10:1) and incubated at 4°C for 30 min. The complexes were subjected to agarose gel electrophoresis to detect the unloaded siRNA.

#### siRNA Stability

The stability of CD44v6-O-MWNTS/Gemcitabine/DOTAP in physical conditions was measured by agarose gel electrophoresis. In brief, free siRNA and CD44v6-O-MWNTS/Gemcitabine/DOTAP/siRNA were suspended in 50% human serum, incubated for 0, 3, 6, 24, 48, and 72 h, followed by separation in a 2% agarose gel to detect the level of siRNA.

### Determination of Encapsulation Efficiency and Percent Drug Loading

The encapsulated CD44v6-O-MWNTS/Gemcitabine/DOTAP were dialyzed (MWCO, 5 kDa) to exclude unencapsulated gemcitabine, freeze-dried, dissolved in methanol, and the amount of gemcitabine was determined by ultraviolet spectrophotometer at 271 nm. The calculation of drug encapsulation efficiency and drug loading capacity (LC) follow the below formulas:

EE% = W_*t*_/W_*O*_ × 100%;

LC% = W_*t*_/W_*S*_ × 100%;

W_*T*_, weight of gemcitabine encapsulated in nanoparticles; W_*O*_, initial weight of gemcitabine; W_*S*_, total weight of the freeze-dried nanoparticles.

### *In vitro* Drug Release

The *in vitro* release of the drug from O-MWNTS/Gemcitabine/DOTAP was assessed by dialysis (Eleraky et al., 2020; Omar et al., 2021). In brief, O-MWNTS/Gemcitabine/DOTAP that contained 3 mg of Gemcitabine were dispersed in 1 mL PBS containing 0.1% Tween-80 (pH 7.4 or pH 6.5) and transferred to the dialysis bag (MWCO, 5 kDa). The dialysis bags were placed into 30 ml of PBS (pH 7.4) with continuous stirring at 37°C and 100 rpm. To determine the level of released drug, 200 μL of media was taken at 0.5, 1, 2, 3, 4, 6, 8, 12, 24, 36, 48, 72, and 96 h, respectively, and the same volume of fresh release media was added. The concentration of released gemcitabine and oxaliplatin in solution was evaluated by ultraviolet spectrophotometer (UV2401, Shimadzu, Japan) at 271 nm and 250 nm, respectively, and the cumulative release amount was calculated. To detect the release of siRNA, O-MWNTS/Gemcitabine/DOTAP/siRNA that contained 2 μg Cy6-labeled siRNA was dispersed in 600 μL PBS at pH 6.5 or 7.4 and incubated at 37°C with stirring. At indicated time points, the samples were centrifuged (13,000 rpm, 30 min), the supernatant was collected for detection of Cy6-labeled siRNA by a microplate reader (PerkinElmer, Germany). The precipitate was resuspended in an equal amount of PBS for further detection.

### Enzymatic Degradation Assay

Free siRNA or equivalent O-MWNTS/Gemcitabine/DOTAP/siRNA were mixed with an equal amount of 50% FBS or 1 μL RNase-A (1 mg/mL) at 37°C. Samples were taken at different time points, immediately frozen at −80°C, and detected by 2% agarose gel electrophoresis.

### Cell Uptake and Mechanisms

To determine cell uptake of the obtained nanocomplexes, CD44v6-O-MWNTS/C6/DOTAP/siRNA was prepared by replacing gemcitabine and oxaliplatin with the fluorescence probe C6. Cy6-labeled siRNA was used for co-localization with C6. SW626 and SKOV-3 cells were seeded in culture plates and were incubated by DMEM solution containing CD44v6-O-MWNTS/C6/DOTAP/siRNA (200 μg/mL) at 37°C for 1, 2, and 4 h, respectively. Next, cells were washed with PBS three times to remove the nanoparticles, fixed with 4% paraformaldehyde (PFA) solution for 15 min, and then rinsed with PBS three times. The uptake of nanoparticles by SW626 cells was observed under a confocal microscope. Similarly, flow cytometry was used to assess the intensity of fluorescence. To investigate the mechanisms related to the uptake of CD44v6-O-MWNTS/C6/DOTAP/siRNA into SW626 and SKOV-3 cells, the inhibitors of clathrin-mediated endocytosis and lipid raft-mediated endocytosis, chlorpromazine hydrochloride, MβCD, and dynasore, were used to treat cells together with administration of CD44v6-O-MWNTS/C6/DOTAP/siRNA.

### Cytotoxicity Test

MTT assay and colony formation assay was used to detect the cytotoxicity of nanocomplexes. For MTT assay, SW626 and SKOV-3 cells were digested, collected, and seeded in 96-well plated at a density of 5,000 cells per well. The indicated nanocomplexes, including O-MWNTS, Gemcitabine, O-MWNTS/Gemcitabine/DOTAP, O-MWNTS/Gemcitabine/DOTAP/siRNA, lipo-siRNA, and CD44v6-O- MWNTS/Gemcitabine/DOTAP/siRNA, were added to the culture medium and incubated for 24 and 48 h. At indicated time point, MTT reagent was added to each well and incubated for another 4 h. Finally, the cell culture medium was discarded and 150 μL DMSO was added to incubate in dark for 15 min. The absorbance values at 490 nm were detected by a microplate reader (PerkinElmer, Waltham, MA, United States).

For the colony formation experiment, SW626 cells were planted in six-well plates (1,000 cells/well), treated with indicated nanocomplexes, and incubated for 2 weeks. The visible clones were fixed by methanol, stained with crystal violate for 20 min, photographed, and counted.

Similar experiments were performed for the detection of cytotoxicity of CD44v6-O- MWNTS/Oxaliplatin/DOTAP/siRNA.

### Western Blotting Experiment

SW626 and SKOV-3 cells were treated with indicated nanocomplexes for 24 h and lysed with ice-cold RIPA solution to extract proteins. The quantification of proteins was conducted by using a BCA kit (Thermo, Waltham, MA, United States). An equivalent 30 μg protein was divided in SDS-PAGE and shifted to NC membranes. The membranes were blocked by fast-blocking reagent (SolarBio, Beijing, China) for 15 min, incubated with primary antibodies against Bax, Bid, Bim, Bcl-2, Caspase-3, Caspase-9, EGFR, CXCR4, and β-actin, at 4°C overnight, followed by incubation with corresponding HRP-conjugated secondary anti-mouse or anti-rabbit antibodies, respectively. The blots were visualized by using ECL reagent (Millipore, Burlington, MA, United States) in a gel imaging system (BD Biosciences, Franklin Lakes, NJ, United States). All antibodies used in this work were purchased from Abcam and diluted according to the manufacturer’s description. Similar experiments were performed for the detection of the cytotoxicity of CD44v6-O-MWNTS/Oxaliplatin/DOTAP/siRNA.

### Detection of Blood Compatibility

Elevated doses of CD44v6-O-MWNTS/Gemcitabine/DOTAP/siRNA (5, 10, 25, 100, and 200 μM) were incubated with red blood cells for 2 h and centrifuged. The heme released by fractured red blood cells to the supernatant was detected by a microplate analyzer at 540 nm.

### *In vivo* Tumor Xenograft and Immunohistochemistry (IHC)

All animal experiments were approved by the Animal Ethics Committee of Cangzhou Central Hospital (Approval no. 2020-120-01). SCID/nude mice (6-week aged) were purchased from Beijing HFK Bioscience (Beijing, China). SW626 cells were inoculated subcutaneously into mice, and 20 nude mice with similar tumor size were selected and randomly divided into five groups with four mice in each group, namely the control, O-MWNTS, Gemcitabine + siRNA, O-MWNTS/Gemcitabine/DOTAP/siRNA, CD44v6-O-MWNTS/Gemcitabine/DOTAP/siRNA. The nanocomplexes were intravenously injected through the tail vein every other day. Tumor size was monitored and body weight was recorded every 3 days. The mice succumbed to death 27 days later, and the tumors and major organs (kidney, liver, and spleen) were isolated for further experiments. The tumor tissues were fixed in 4% PFA, dehydrated, embedded in paraffin, and sliced into 5 μM thick sections. The tumor sections were incubated with primary antibodies against Ki67 and CXCR4 antibody overnight at 4°C, followed by incubation with biotin-conjugated secondary antibodies for 1 h at room temperature. Hematoxylin-eosin (HE) staining was conducted to evaluate tissue damage. The positive staining was quantitatively analyzed using ImageJ software. Similar experiments were performed for *in vivo* evaluation of CD44v6-O- MWNTS/Oxaliplatin/DOTAP/siRNA.

### Statistics

All data in this work are provided as means ± SD, and analyzed by Student’s *t*-test or one-way ANOVA method in SPSS software (Version 19.0). *p* < 0.05 was considered significant.

## Results

### CD44v6-O-MWNTS/Gemcitabine/DOTAP Preparation and Characterization

**FIGURE 1:**
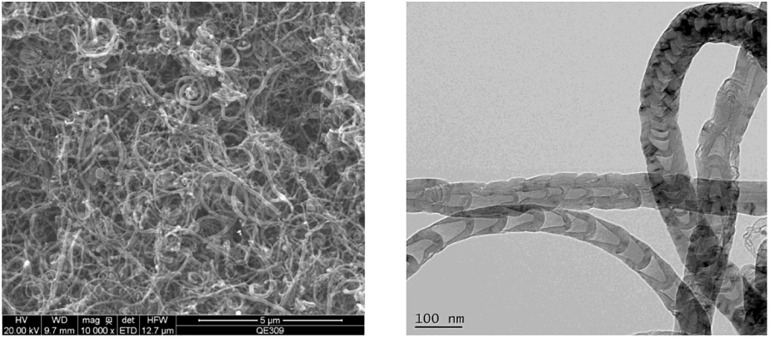
CD44v6-O-MWNTS/Gemcitabine/DOTAP preparation and characterization. The scanning electron microscope (SEM) (left) image and transmission electron microscope (TEM) (right) image of CD44v6-O-MWNTS/Gemcitabine/DOTAP.

Firstly, the morphology of pristine MWNTs (Supplementary Figure 1A) was detected by TEM, and the successful synthesis of O-MWNTS was manifested by a higher intensity of D band (1330 nm) than G band (1590 nm) of O-MWNTs determined by Raman spectra (Supplementary Figure 1B). The characteristic peaks of -CONH- were observed in nuclear magnetic resonance (NMR) spectra of CD44v6-O-MWNTS (Supplementary Figure 1C), which was also confirmed by results of Fourier transform infrared spectroscopy (FTIR, Supplementary Figure 1D) and Raman spectra (Supplementary Figure 1E). Subsequently, CD44v6-O-MWNTS/Gemcitabine/DOTAP was successfully synthesized and characterized by transmission electron microscope (TEM) and scanning electron microscope (SEM). As shown in Figure 1, the TEM and SEM image manifested the complex structure of carbon tubes, the diameter of the CD44v6-O-MWNTS/Gemcitabine/DOTAP was around 70 nm, and length was at the micrometer scale.

CD44v6-O-MWNTs-Gemcitabine/DOTAP (201 ± 3.19 nm) showed a larger particle size than O-MWNTs (150 ± 1.27 nm) (Supplementary Table 1). The higher polydispersity index (PDI) value of CD44v6-O-MWNTs-Gemcitabine/DOTAP also suggested its larger particle size. Besides, the positive zeta potential of CD44v6-O-MWNTs-Gemcitabine/DOTAP demonstrated a stronger ability to penetrate the cell membrane (Supplementary Table 1). The drug loading capacity (LC%) and entrapment efficiency (EE%) of CD44v6-O-MWNTs-Gemcitabine/DOTAP were around 16 and 87% separately, manifested the satisfactory effectiveness as a drug delivery system (Supplementary Table 2).

### Assessment of siRNA-Loading Capacity and Drug Release

**FIGURE 2:**
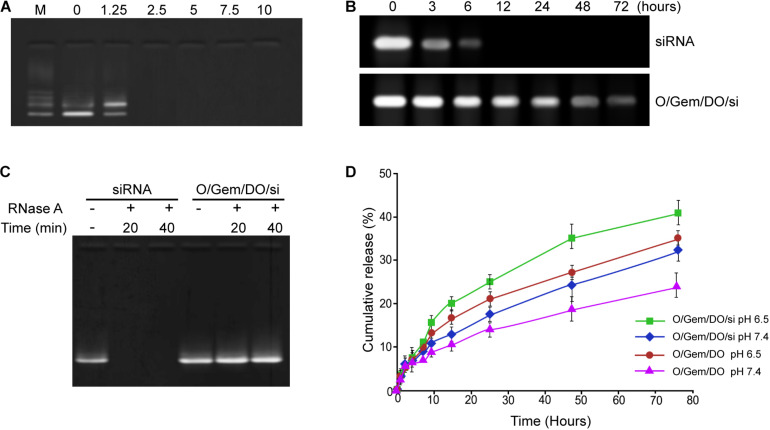
Assessment of siRNA-loading capacity and release. **(A)** The siRNA loading ability under diverse ratio of siRNA and CD44v6-O-MWNTS/Gemcitabine/DOTAP was detected by agarose gel electrophoresis. **(B,C)** The stability of CD44v6-O-MWNTS/Gemcitabine/DOTAP/siRNA in RNase A **(B)** and serum **(C)** was analyzed by agarose gel electrophoresis. **(D)** The drug release analysis was performed at pH 6.5 and pH 7.4. Release of gemcitabine and siRNA was detected by ultraviolet spectrophotometer. O/Gem/DO: O-MWNTS/Gemcitabine/DOTAP; O/Gem/DO/si: O-MWNTS/Gemcitabine/DOTAP/siRNA.

The siRNA-loading capacity was analyzed by agarose gel electrophoresis. The bands of free siRNA disappeared when the ratio was 1:2.5 and above, indicating that CD44v6-O-MWNTS/Gemcitabine/DOTAP was able to fully load siRNA at this ratio (Figure 2A). Meanwhile, the stability analysis showed that the free siRNA was quickly and completely degraded in the presence of RNase A, while O- MWNTS/Gemcitabine/DOTAP/siRNA still had intact siRNA bands after co-incubation with RNase A, indicating that the carbon nanotubes could protect the siRNA (Figure 2B). Moreover, the siRNA alone was notably degraded within 6 h after incubating in 50% serum, while the siRNAs loaded in O-MWNTS/Gemcitabine/DOTAP were stable even after 48-h incubation (Figure 2C). The drug release analysis showed that O-MWNTS/Gemcitabine/DOTAP, O- MWNTS/Gemcitabine/DOTAP/siRNA, O-MWNTS/Oxaliplatin/DOTAP, O-MWNTS/Oxaliplatin/DOTAP/siRNA were able to effectively release the siRNA, gemcitabine, and oxaliplatin in a time-dependent manner within 80 h (Figure 2D and Supplementary Figure 1F), especially at pH 6.5, which fits the acid condition in tumor sites.

### The Cellular Uptake of CD44v6-O-MWNTS/Drug/DOTAP/siRNA

**FIGURE 3:**
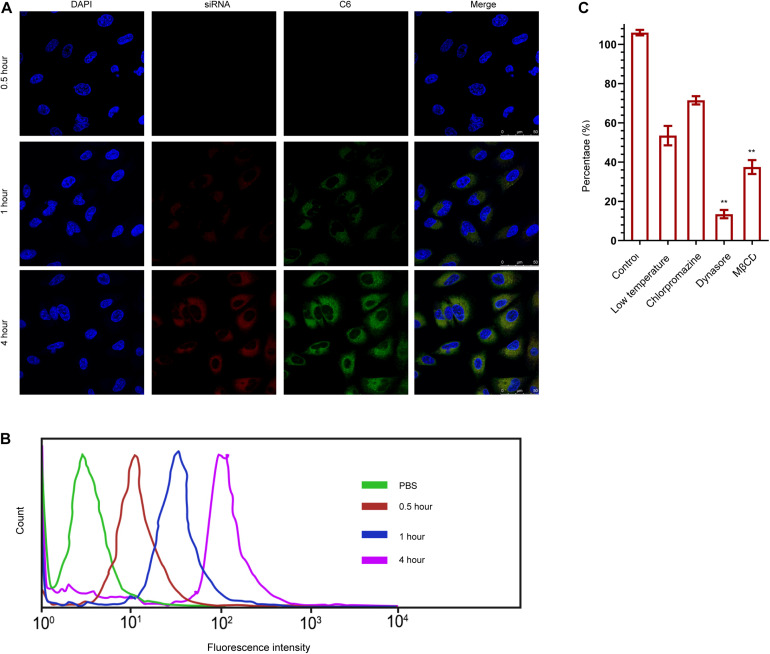
The cellular uptake of CD44v6-O-MWNTS/Gemcitabine/DOTAP/siRNA by SW626. **(A,B)** The cellular uptake of CD44v6-O-MWNTS/Gemcitabine/DOTAP/siRNA was analyzed by confocal fluorescence microscope **(A)** and flow cytometry **(B)** in SW626 cells. Fluorescence probe C6 was used to replace gemcitabine in CD44v6-O-MWNTS/C6/DOTAP/siRNA system for localization, and siRNA was labeled by Cy6 fluorescence probe. Five random areas were captured. **(C)** Cells were treated with low temperature, chlorpromazine hydrochloride [inhibitor of lattice (clathrin)-mediated endocytosis] or MβCD and dynasore [inhibitor of lipid-raft (lipid-raft)-mediated endocytosis]. The cellular uptake mechanism of CD44v6-O-MWNTS/Gemcitabine/DOTAP/siRNA was examined by intracellular drug concentration. **P < 0.01.

The cellular uptake of O-MWNTS/Gemcitabine/DOTAP/siRNA was then analyzed by confocal fluorescence microscope and flow cytometry in ovarian cancer cells. Fluorescence probe C6 was used to replace gemcitabine in CD44v6-O-MWNTS/C6/DOTAP/siRNA system, and siRNA was labeled by Cy6 fluorescence probe. The co-localization of Cy6-labeled siRNA and C6 in the cytoplasm of SW626 within 4 h after incubation was detected by confocal (Figure 3A), which demonstrated that O-MWNTS/Gemcitabine/DOTAP/siRNA was able to be effectively uptake by SW626 cells. Moreover, results from flow cytometry also indicated enhanced intensity of fluorescence, namely internalization of drugs and siRNAs in SW626 and SKOV-3 cells (Figure 3B and Supplementary Figure 2A).

We then investigated the mechanism of CD44v6-O-MWNTS/C6/DOTAP/siRNA entering into the cell by inhibiting the typical endocytic pathway. All active transport needed energy participation, and we first investigated by low-temperature treatment whether CD44v6-O-MWNTS/C6/DOTAP/siRNA entry into the cells by active transport. The endocytic pathway of cells mainly consisted of lattice (clathrin)-mediated endocytosis and lipid-raft (lipid-raft)-mediated endocytosis. The inhibitors of the former were chlorpromazine hydrochloride and the inhibitors of the latter were MβCD and dynasore. The uptake of CD44v6-O-MWNTS/C6/DOTAP/siRNA without any inhibitor was used as a control. We identified that endocytosis of CD44v6-O-MWNTS/C6/DOTAP/siRNA was mainly dependent on lipid raft-mediated endocytosis, and the relevant inhibitors significantly reduced the intracellular drug concentration in SW626 MG (Figure 3C) and SKOV-3 cells (Supplementary Figure 2B).

### The Effect of CD44v6-O-MWNTS/Drug/DOTAP/siRNA on Ovarian Cancer Cell Proliferation and Apoptosis *in vitro*

**FIGURE 4:**
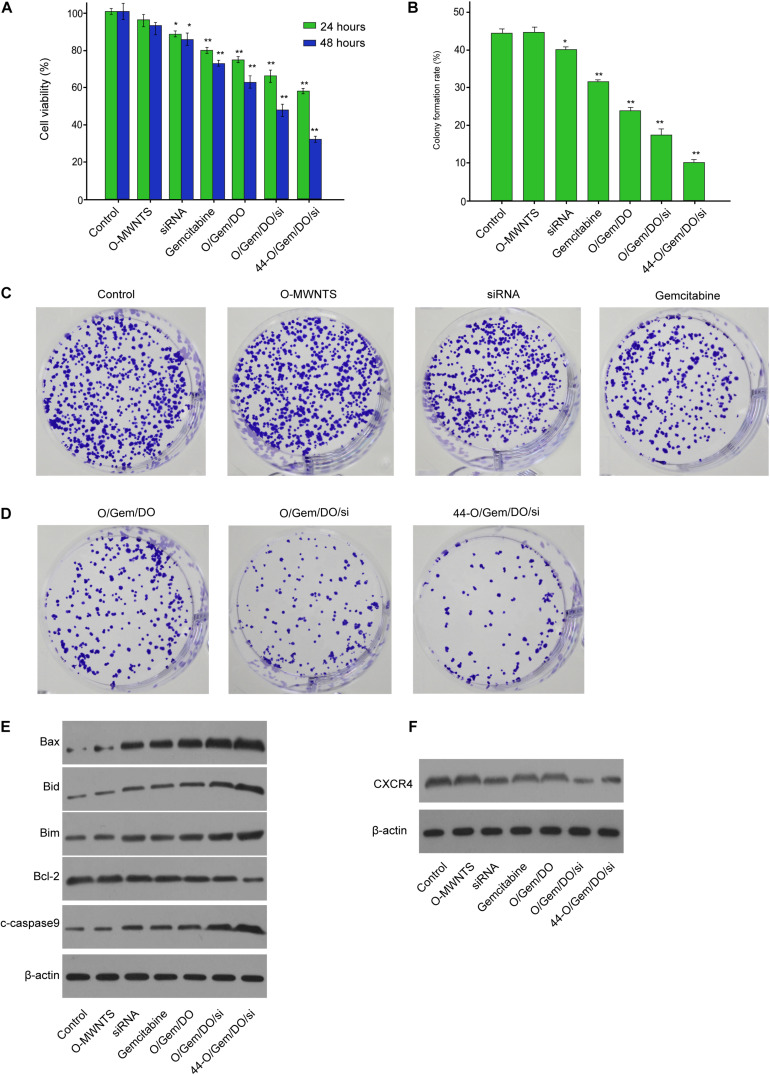
The effect of CD44v6-O-MWNTS/Gemcitabine/DOTAP/siRNA on SW626 cancer cell proliferation and apoptosis *in vitro*. **(A–E)** The SW626 cells were treated as the indicated labeling. **(A)** The cell viability was analyzed by MTT assays. **(B,C)** The cell proliferation was measured by colony formation assays. **(D)** The expression of Bax, Bid, Bim, Bcl-2, cleaved caspase-9 (c-caspase-9) was measured by Western blot analysis. **(F)** The expression of CXCR4 was detected by Western blot analysis. O/Gem/DO: O-MWNTS/Gemcitabine/DOTAP; O/Gem/DO/si: O-MWNTS/Gemcitabine/DOTAP/siRNA; 44-O/Gem/DO/si: CD44v6-O-MWNTS/Gemcitabine/DOTAP/siRNA. Data are presented as mean ± SD. Statistic significant differences were indicated: *P < 0.05, **P < 0.01.

We then found that CD44v6-O-MWNTS/Gemcitabine/DOTAP/siRNA inhibited the SW626 and SKOV-3 cell viability compared with O-MWNTS, siRNA, Gemcitabine, MWNTS/Gemcitabine/DOTAP, and O-MWNTS/Gemcitabine/DOTAP/siRNA (Figure 4A and Supplementary Figure 2C). The colony formation numbers of SW626 (Figures 4B,C) and SKOV-3 (Supplementary Figure 2D) cells were notably reduced by CD44v6-O-MWNTS/Gemcitabine/DOTAP/siRNA. The treatment of CD44v6-O-MWNTS/Gemcitabine/DOTAP/siRNA enhanced Bax, Bid, Bim, cleaved caspase-9 expression and reduced Bcl-2 expression in SW626 cells (Figure 4D) and SKOV-3 cells (Supplementary Figure 2E). Besides, the expression of CXCR4 was decreased by CD44v6-O-MWNTS/Gemcitabine/DOTAP/siRNA in SW626 cells (Figure 4E) and SKOV-3 cells (Supplementary Figure 2F). Moreover, CD44v6-O-MWNTS/Oxaliplatin/DOTAP/siRNA also notably reduced proliferative ability and elevated apoptotic signaling proteins in both SW626 and SKOV-3 cells (Supplementary Figure 3).

### The Effect of CD44v6-O-MWNTS/Drug/DOTAP/siRNA on Ovarian Cancer Cell Growth *in vivo*

**FIGURE 5:**
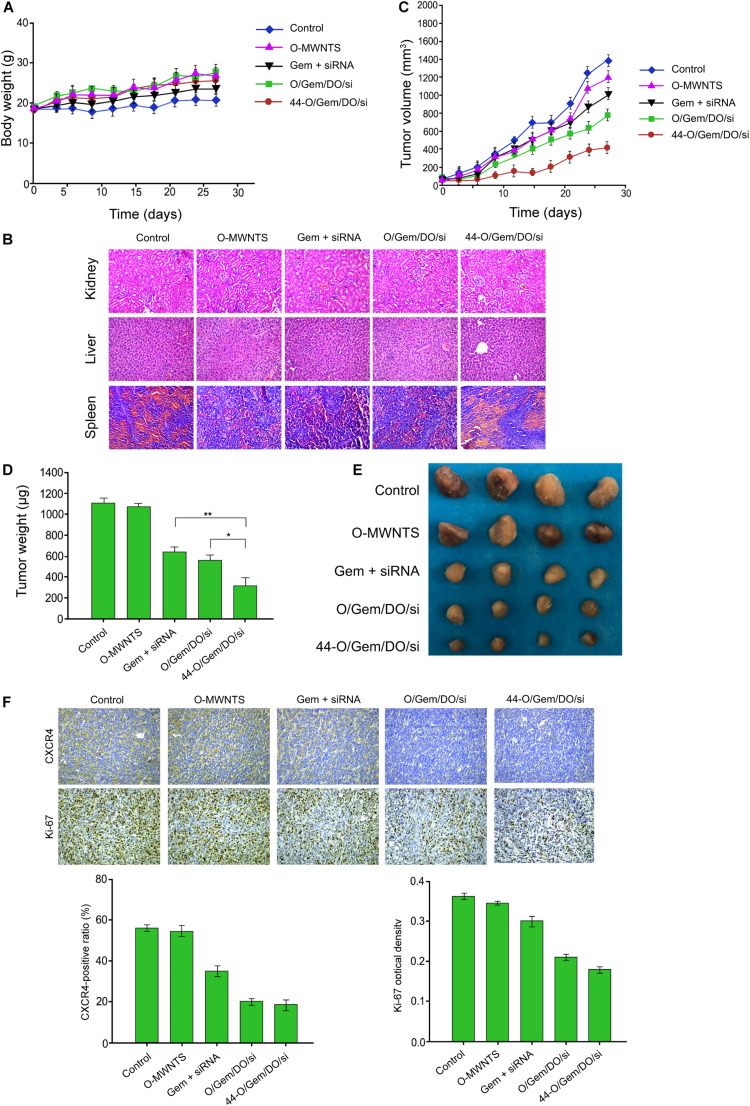
The effect of CD44v6-O-MWNTS/Gemcitabine/DOTAP/siRNA on ovarian cancer cell growth *in vivo*. **(A–E)** The nude mice were injected with SW626 cells and were treated as the indicated labeling. **(A)** The body weight was remarketed. **(B)** HE staining of kidney, liver, and spleen. **(C)** The tumor growth curve was shown. **(D)** The tumor weight was shown. **(E)** The tumor size was shown. **(F)** The levels of Ki-67 and CXCR4 were measured by IHC. Gem: gemcitabine; O/Gem/DO/si: O-MWNTS/Gemcitabine/DOTAP/siRNA; 44-O/Gem/DO/si: CD44v6-O-MWNTS/Gemcitabine/DOTAP/siRNA. Data are presented as mean ± SD. Statistic significant differences were indicated: *P < 0.05, **P < 0.01.

We then determined the effect of CD44v6-O-MWNTS/Drug/DOTAP/siRNA on ovarian cancer cell growth *in vivo*. Tumorigenicity analysis in nude mice showed that CD44v6-O-MWNTS/Gemcitabine/DOTAP/siRNA did not affect the body weight of the mice (Figure 5A), and displayed no damage on normal tissues including kidney, liver, and spleen, as was indicated by HE staining (Figure 5B). CD44v6-O-MWNTS/Gemcitabine/DOTAP/siRNA attenuated the tumor volume, tumor weight, and tumor size in the nude mice compared with O-MWNTS, siRNA + Gemcitabine, and O-MWNTS/Gemcitabine/DOTAP/siRNA (Figures 5C–E). CD44v6-O-MWNTS/Oxaliplatin/DOTAP/siRNA showed similar effects on inhibiting tumor growth (Supplementary Figures 4A–C). Meanwhile, the levels of Ki-67 and CXCR4 were repressed by CD44v6-O-MWNTS/Gemcitabine/DOTAP/siRNA (Figure 5F) and CD44v6-O-MWNTS/Oxaliplatin/DOTAP/siRNA in the system (Supplementary Figure 4D).

## Discussion

Ovarian cancer is the most aggressive subtype of female cancer that seriously threatens women’s health. Carbon nanotubes have great potential for the targeted therapy of tumors. CXCR4 is closely related to the migration, invasion, and metastasis of ovarian cancers. In this study, we applied small interfering RNA targeting the CXCR4 gene and the clinical treatment gemcitabine of ovarian cancer as the therapeutic drug, and organically integrate chemotherapy and gene therapy through carbon nanotubes, and use CD44v6 single chain antibody as the targeting moiety to explore its application in ovarian cancer treatment. We identified that CD44v6-O-MWNTS/Gemcitabine/DOTAP/siRNA demonstrated a remarkable inhibitory effect on ovarian cancers *in vitro* and *in vivo*.

O-MWNTS, DOTAP, and CD44v6 have been broadly used in drug delivery for cancer treatment. It has been reported that DOTAP is applied in PEGylated oxidized multi-walled carbon nanotubes modified with angiopep-2 for the treatment of glioma (Omar et al., 2021). Targeted delivery of doxorubicin by aptamer functionalized DOTAP/DOPE liposomes represses breast cancer progression (Eleraky et al., 2020). MWNTs-Fe3O4 nanomaterials inhibit human U87 tumors *in vivo* photothermal treatment (Ren et al., 2012). CD44v6 monoclonal antibody-conjugated gold nanostars target plasmonic photothermal therapy of gastric cancer stem-like cells (Shen et al., 2013). It has also been reported that the inhibition of the CXCL12/CXCR4 pathways improves survival in ovarian cancer cells by preventing immunosuppression (Liang et al., 2015). The notch pathway contributes to the migration and proliferation of ovarian cancer cells by the CXCR4/SDF1α chemokine system (Song et al., 2015). The CXCL12/CXCR4 pathway enhances metastasis, invasion, migration, and the proliferation of ovarian cancer (Guo et al., 2014; Chiaramonte et al., 2015; Zeng et al., 2019).

In this study, we successfully constructed a CD44v6-O-MWNTS/Gemcitabine/DOTAP/siRNA system and a CD44v6-O-MWNTS/Oxaliplatin/DOTAP/siRNA system. O-MWNTS/Gemcitabine/DOTAP/siRNA and CD44v6-O-MWNTS/Oxaliplatin/DOTAP/siRNA system were able to effectively release the Gemcitabine and Oxaliplatin in a time-dependent manner and were associated with pH. The sustained release of drug and siRNA for a long duration facilitates its accumulation in tumor sites and cytotoxicity, and the better release effectiveness under acid conditions fits the acidic environment of tumor sites. The kinetic of drug release may be associated with the interaction between hydrophobic drugs and carbon nanotubes. The cellular uptake of CD44v6-O-MWNTS/Gemcitabine/DOTAP/siRNA was mainly dependent on lipid raft-mediated endocytosis. It indicates a novel delivery system for CXCR4 siRNA and provides a new strategy for targeting cancer cells by the Nano system. Meanwhile, we found that CD44v6-O-MWNTS/Gemcitabine/DOTAP/siRNA improved the effect of siRNA on the inhibition of ovarian cancer cell viability and the induction of cell apoptosis. Tumorigenicity analysis in nude mice showed that CD44v6-O-MWNTS/Gemcitabine/DOTAP/siRNA and CD44v6-O-MWNTS/Oxaliplatin/DOTAP/siRNA significantly repress the tumor growth of ovarian cancer cells *in vivo*. The levels of Ki-67 and CXCR4 were repressed by CD44v6-O-MWNTS/Gemcitabine/DOTAP/siRNA in the system. Our data suggest that CD44v6-O-MWNTS/DOTAP could significantly improve the delivery and uptake effectiveness of CXCR4 siRNA and clinical drug including Gemcitabine and Oxaliplatin for the treatment of ovarian cancer.

We thus concluded that the obtained CD44v6-O-MWNTS could effectively load drugs and CXCR4 siRNA, internalized by cancer cells and realized notable *in vitro* and *in vivo* inhibitory function against ovarian cancer growth. Our study provided a promising nanomaterial for the co-delivery of siRNA and anti-tumor drugs for the therapy of ovarian cancer.

## Data Availability Statement

The original contributions presented in the study are included in the article/Supplementary Material, further inquiries can be directed to the corresponding author/s.

## Ethics Statement

The animal study was reviewed and approved by the Cangzhou Central Hospital.

## Author Contributions

WY designed and performed the experiments. S–MQ collected and analyzed the data and wrote the manuscript. Both authors contributed to the article and approved the submitted version.

## Conflict of Interest

The authors declare that the research was conducted in the absence of any commercial or financial relationships that could be construed as a potential conflict of interest.

## Supplementary Material

Supplementary Figure 1 Characterization of CD44v6-O-MWNTS system. **(A)** TEM image of pristine MWNTs. **(B)** The Raman spectra of O-MWNTS. **(C–E)** The nuclear magnetic resonance (NMR) spectra **(C)**, Fourier transform infrared spectroscopy (FTIR, **D**), and Raman spectra **(E)** of CD44v6-O-MWNTs. **(F)** The release of Oxaliplatin and siRNA was detected by ultraviolet spectrophotometer at pH 6.5 and pH 7.4. O/Oxa/DO: O-MWNTS/Oxaliplatin/DOTAP; O/Oxa/DO/si: O-MWNTS/Oxaliplatin/DOTAP/siRNA.

Supplementary Figure 2 The effect of CD44v6-O-MWNTS/Gemcitabine/DOTAP/siRNA on SKOV-3 cancer cells. **(A)** The cellular uptake of CD44v6-O- MWNTS/Gemcitabine/DOTAP/siRNA by SKOV-3 cells were analyzed by flow cytometry. **(B)** The cellular uptake mechanism was examined. **(C–F)** The SKOV-3 cells were treated as the indicated labeling. The cell viability was analyzed by MTT assays **(C)**. The cell proliferation was measured by colony formation assays **(D)**. The expression of Bax, Bid, Bim, Bcl-2, cleaved caspase-9 (c-caspase-9) was measured by Western blot analysis **(E)**. **(F)** The expression of CXCR4 was detected by Western blot analysis. O/Gem/DO: O-MWNTS/Gemcitabine/DOTAP; O/Gem/DO/si: O-MWNTS/Gemcitabine/DOTAP/siRNA; 44-O/Gem/DO/si: CD44v6-O-MWNTS/Gemcitabine/DOTAP/siRNA. Data are presented as mean ± SD. Statistic significant differences were indicated: **P* < 0.05, ***P* < 0.01.

Supplementary Figure 3 The effect of CD44v6-O-MWNTS/Oxaliplatin/DOTAP/siRNA on ovarian cancer cell proliferation and apoptosis *in vitro*. **(A–D)** The SW626 cells were treated as the indicated labeling. **(A)** The cell viability was analyzed by MTT assays. **(B)** The cell proliferation was measured by colony formation assays. **(C)** The expression of Bax, Bid, Bim, Bcl-2, cleaved caspase-9 (c-caspase-9) was measured by Western blot analysis. **(D)** The expression of CXCR4 was detected by Western blot analysis. **(E–H)** The SKOV-3 cells were treated as the indicated labeling. The cell viability was analyzed by MTT assays **(E)**. The cell proliferation was measured by colony formation assays **(F)**. **(G)** The expression of Bax, Bid, Bim, Bcl-2, cleaved caspase-9 (c-caspase-9) was measured by Western blot analysis. **(H)** The expression of CXCR4 was detected by Western blot analysis. Oxa: oxaliplatin; O/Oxa/DO: O-MWNTS/Oxaliplatin/DOTAP; O/Oxa/DO/si: O-MWNTS/Oxaliplatin/DOTAP/siRNA; 44-O/Oxa/DO/si: CD44v6-O-MWNTS/Oxaliplatin/DOTAP/siRNA. Data are presented as mean ± SD. Statistic significant differences were indicated: **P* < 0.05, ***P* < 0.01.

Supplementary Figure 4 The effect of CD44v6-O-MWNTS/Oxaliplatin/DOTAP/siRNA on ovarian cancer cell growth *in vivo*. The nude mice were injected with SW626 cells and were treated as the indicated labeling. **(A)** The body weight was remarketed. **(B)** The tumor volume was shown. **(C)** The tumor size and weight were shown. **(D)** The levels of Ki-67 and CXCR4 were measured by IHC. Oxa: oxaliplatin; O/Oxa/DO/si: O-MWNTS/Oxaliplatin/DOTAP/siRNA; 44-O/Oxa/DO/si: CD44v6-O-MWNTS/Oxaliplatin/DOTAP/siRNA**.** Data are presented as mean ± SD. Statistic significant differences were indicated: **P* < 0.05, ***P* < 0.01.
