# Supplementary material for: CD44v6-O-MWNTS-Loaded Gemcitabine and CXCR4 siRNA Improves the Anti-tumor Effectiveness of Ovarian Cancer
Source: Front Cell Dev Biol. 2021 Jul 7;9:687322. doi: 10.3389/fcell.2021.687322 (PMC8292962; doi:10.3389/fcell.2021.687322)
Supplement: Supplementary file 1 [file Table_1.DOCX]

Table S1 Characterization of nanotubes

| Formulation | Particle size (nm) | PDI | zeta potential |
| --- | --- | --- | --- |
| O-MWNTS | 150 ± 1.27 | 0.239 ± 0.005 | -18.83 ± 0.62 |
| CD44v6-O-MWNTS/Gemcitabine/DOTAP | 201 ± 3.19 | 0.371 ± 0.013 | 23.5 ± 0.21 |
