# Supplementary material for: CD44v6-O-MWNTS-Loaded Gemcitabine and CXCR4 siRNA Improves the Anti-tumor Effectiveness of Ovarian Cancer
Source: Front Cell Dev Biol. 2021 Jul 7;9:687322. doi: 10.3389/fcell.2021.687322 (PMC8292962; doi:10.3389/fcell.2021.687322)
Supplement: Supplementary file 2 [file Table_2.DOCX]

Table S2 characterization of encapsulation efficiency EE%) and percent drug loading capacity (LC%)

| Formulation | EE% | LC% |
| --- | --- | --- |
| CD44v6-O-MWNTS/Gemcitabine/DOTAP | 87 ± 0.19 | 16.48 ± 0.1 |
