# Supplementary figures and images for: CD44v6-O-MWNTS-Loaded Gemcitabine and CXCR4 siRNA Improves the Anti-tumor Effectiveness of Ovarian Cancer
Source: Front Cell Dev Biol. 2021 Jul 7;9:687322. doi: 10.3389/fcell.2021.687322 (PMC8292962; doi:10.3389/fcell.2021.687322)

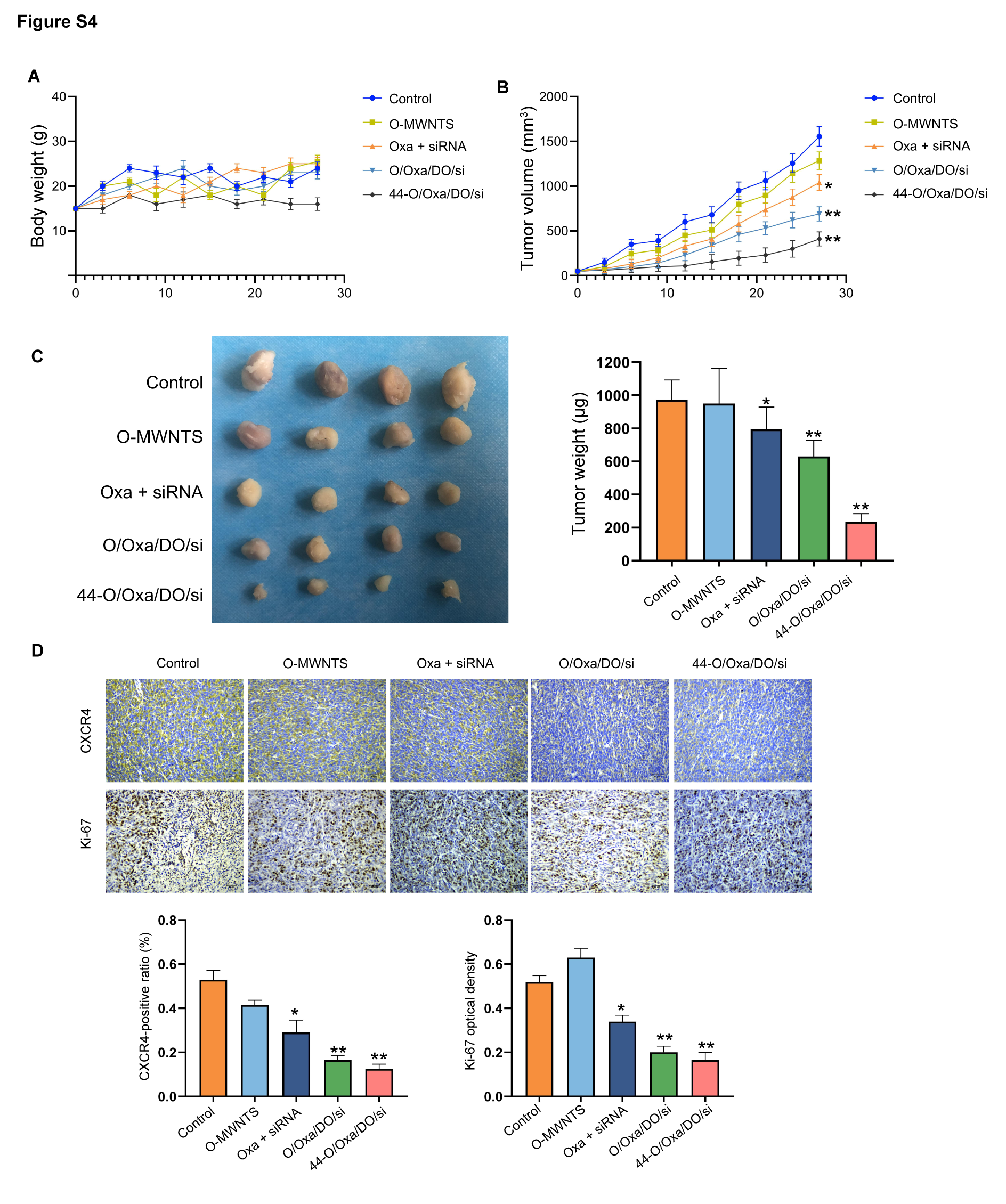

Supplement: Supplementary Figure 1 — Characterization of CD44v6-O-MWNTS system. (A) TEM image of pristine MWNTs. (B) The Raman spectra of O-MWNTS. (C–E) The nuclear magnetic resonance (NMR) spectra (C), Fourier transform infrared spectroscopy (FTIR, D), and Raman spectra (E) of CD44v6-O-MWNTs. (F) The release of Oxaliplatin and siRNA was detected by ultraviolet spectrophotometer at pH 6.5 and pH 7.4. O/Oxa/DO: O-MWNTS/Oxaliplatin/DOTAP; O/Oxa/DO/si: O-MWNTS/Oxaliplatin/DOTAP/siRNA. [file Image_1.tif]

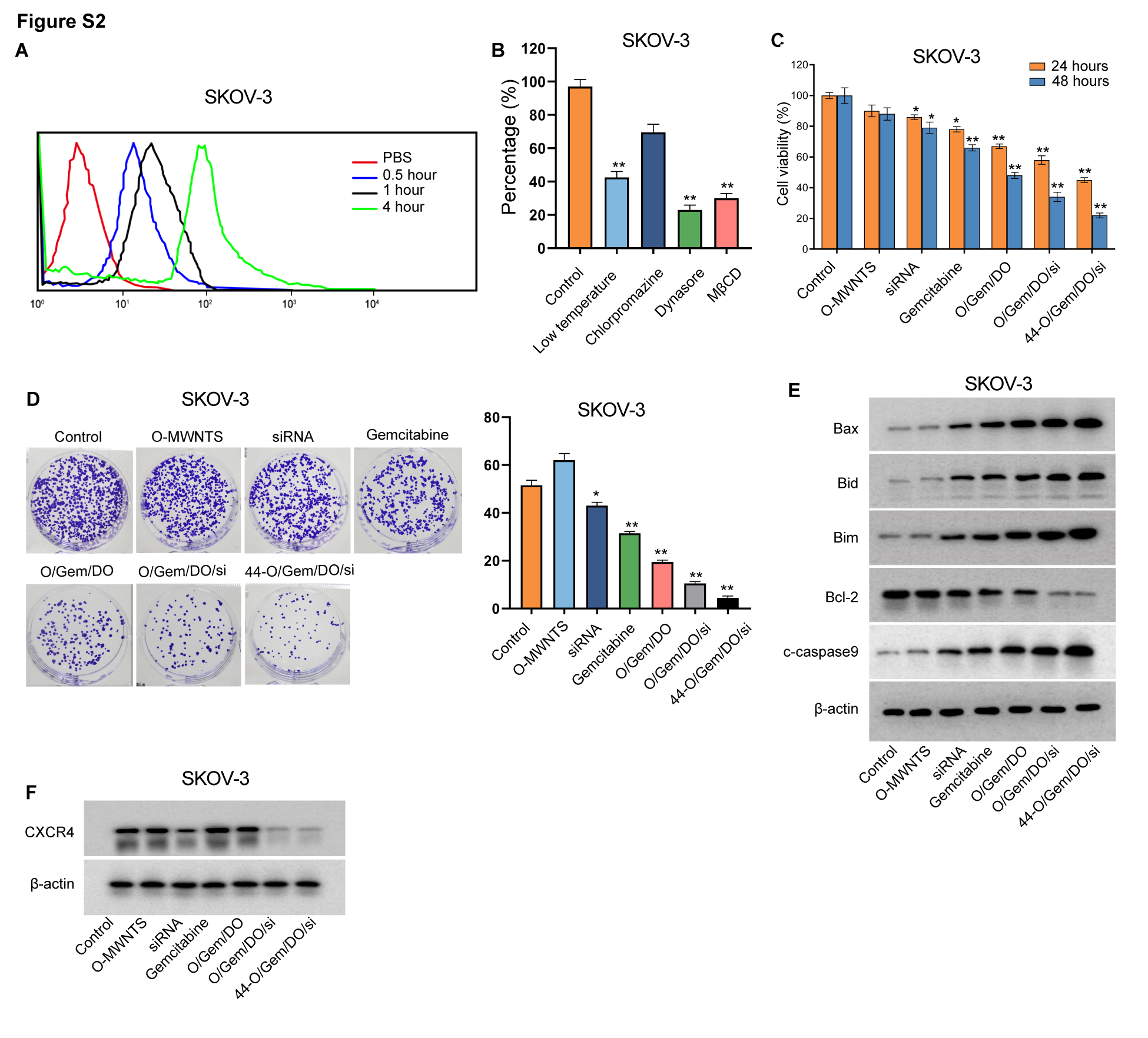

Supplement: Supplementary Figure 2 — The effect of CD44v6-O-MWNTS/Gemcitabine/DOTAP/siRNA on SKOV-3 cancer cells. (A) The cellular uptake of CD44v6-O- MWNTS/Gemcitabine/DOTAP/siRNA by SKOV-3 cells were analyzed by flow cytometry. (B) The cellular uptake mechanism was examined. (C–F) The SKOV-3 cells were treated as the indicated labeling. The cell viability was analyzed by MTT assays (C). The cell proliferation was measured by colony formation assays (D). The expression of Bax, Bid, Bim, Bcl-2, cleaved caspase-9 (c-caspase-9) was measured by Western blot analysis (E). (F) The expression of CXCR4 was detected by Western blot analysis. O/Gem/DO: O-MWNTS/Gemcitabine/DOTAP; O/Gem/DO/si: O-MWNTS/Gemcitabine/DOTAP/siRNA; 44-O/Gem/DO/si: CD44v6-O-MWNTS/Gemcitabine/DOTAP/siRNA. Data are presented as mean ± SD. Statistic significant differences were indicated: *P < 0.05, **P < 0.01. [file Image_2.TIF]

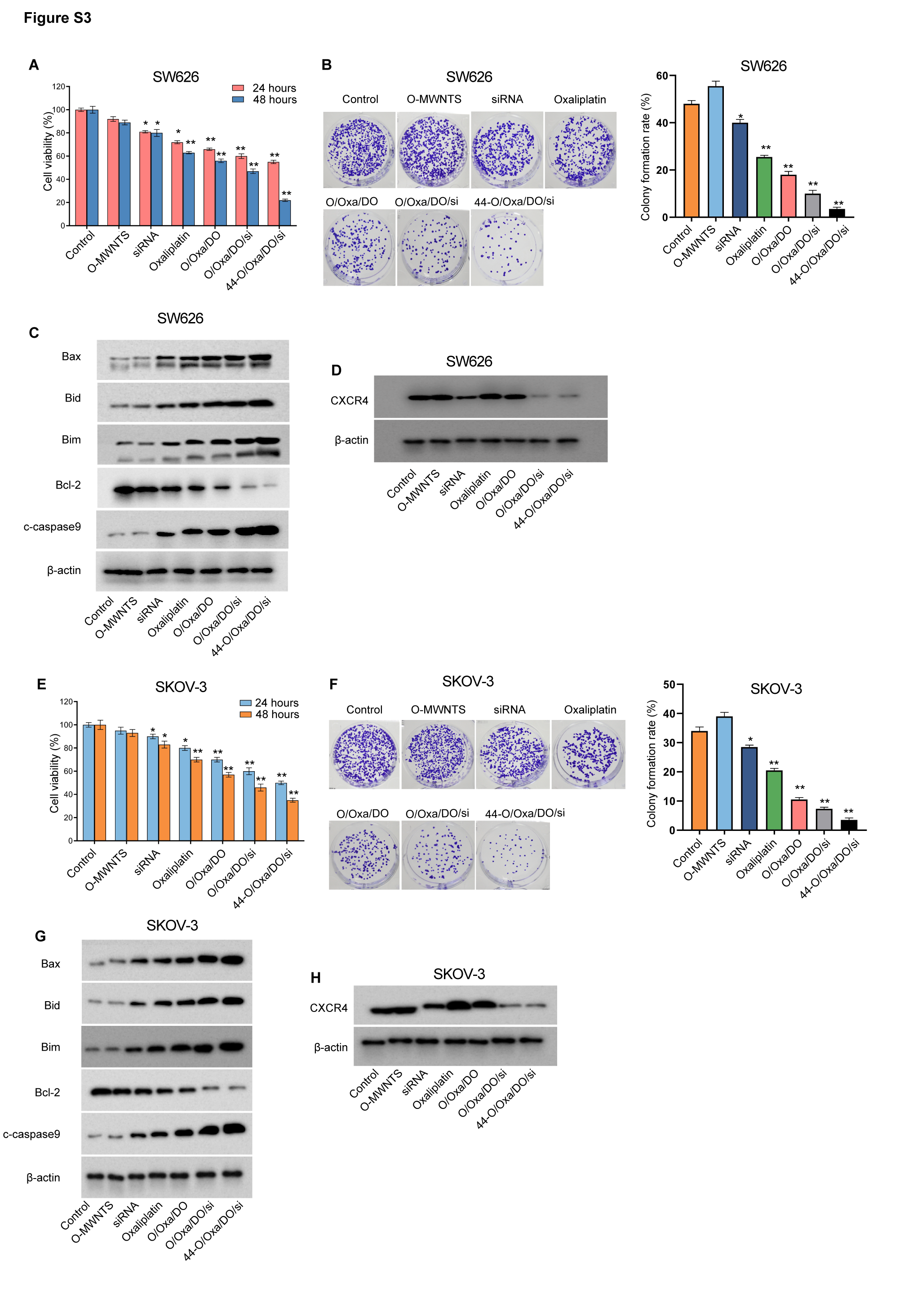

Supplement: Supplementary Figure 3 — The effect of CD44v6-O-MWNTS/Oxaliplatin/DOTAP/siRNA on ovarian cancer cell proliferation and apoptosis in vitro. (A–D) The SW626 cells were treated as the indicated labeling. (A) The cell viability was analyzed by MTT assays. (B) The cell proliferation was measured by colony formation assays. (C) The expression of Bax, Bid, Bim, Bcl-2, cleaved caspase-9 (c-caspase-9) was measured by Western blot analysis. (D) The expression of CXCR4 was detected by Western blot analysis. (E–H) The SKOV-3 cells were treated as the indicated labeling. The cell viability was analyzed by MTT assays (E). The cell proliferation was measured by colony formation assays (F). (G) The expression of Bax, Bid, Bim, Bcl-2, cleaved caspase-9 (c-caspase-9) was measured by Western blot analysis. (H) The expression of CXCR4 was detected by Western blot analysis. Oxa: oxaliplatin; O/Oxa/DO: O-MWNTS/Oxaliplatin/DOTAP; O/Oxa/DO/si: O-MWNTS/Oxaliplatin/DOTAP/siRNA; 44-O/Oxa/DO/si: CD44v6-O-MWNTS/Oxaliplatin/DOTAP/siRNA. Data are presented as mean ± SD. Statistic significant differences were indicated: *P < 0.05, **P < 0.01. [file Image_3.TIF]
